# Supplementary figures and images for: Haploinsufficiency by minute MutL homolog 1 promoter DNA methylation may represent unique phenotypes of microsatellite instability-gastric carcinogenesis
Source: PLoS One. 2021 Dec 22;16(12):e0260303. doi: 10.1371/journal.pone.0260303 (PMC8694418; doi:10.1371/journal.pone.0260303)

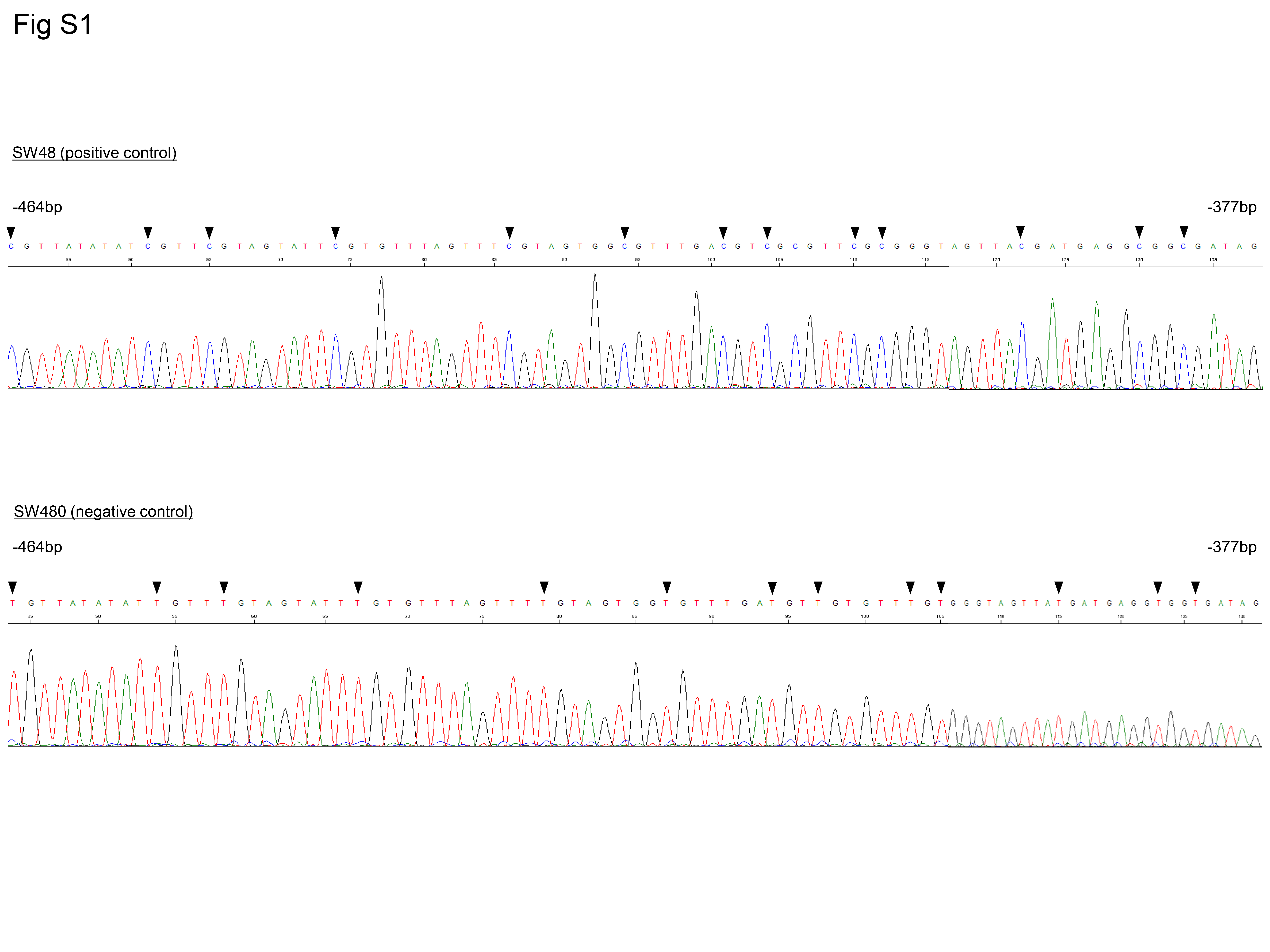

Supplement: S1 Fig — (TIF) [file pone.0260303.s001.tif]

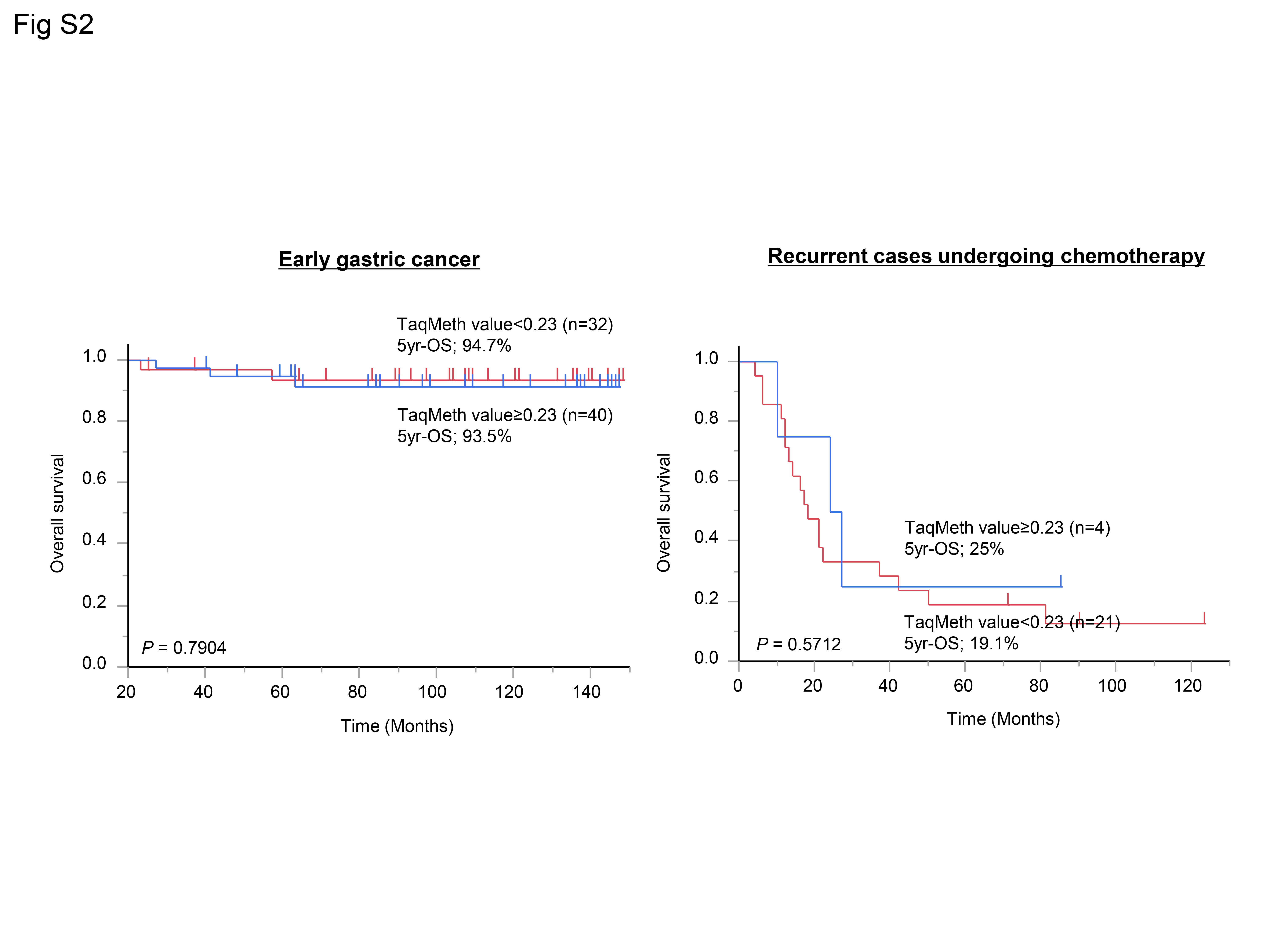

Supplement: S2 Fig — (TIF) [file pone.0260303.s002.tif]

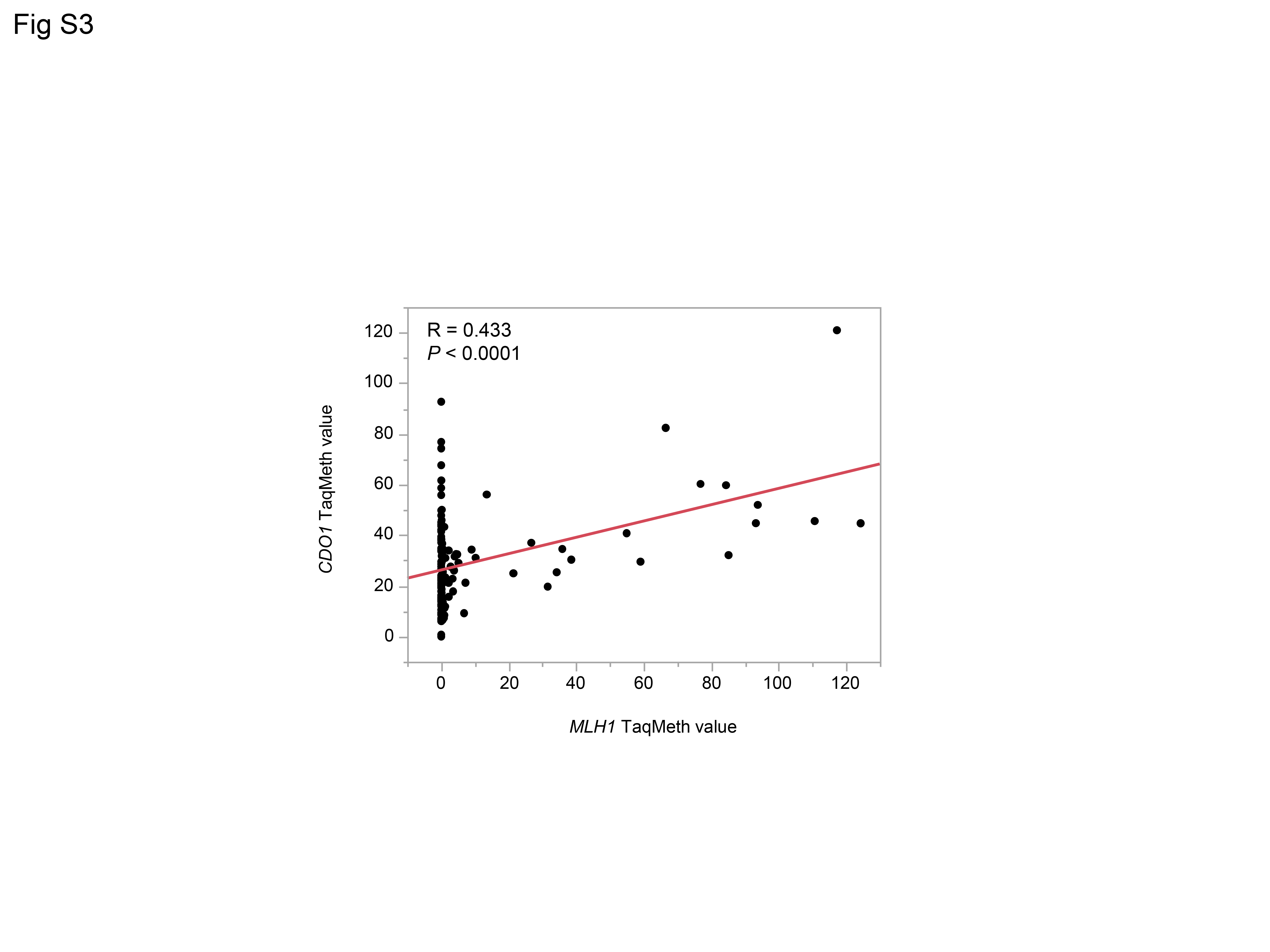

Supplement: S3 Fig — (TIF) [file pone.0260303.s003.tif]

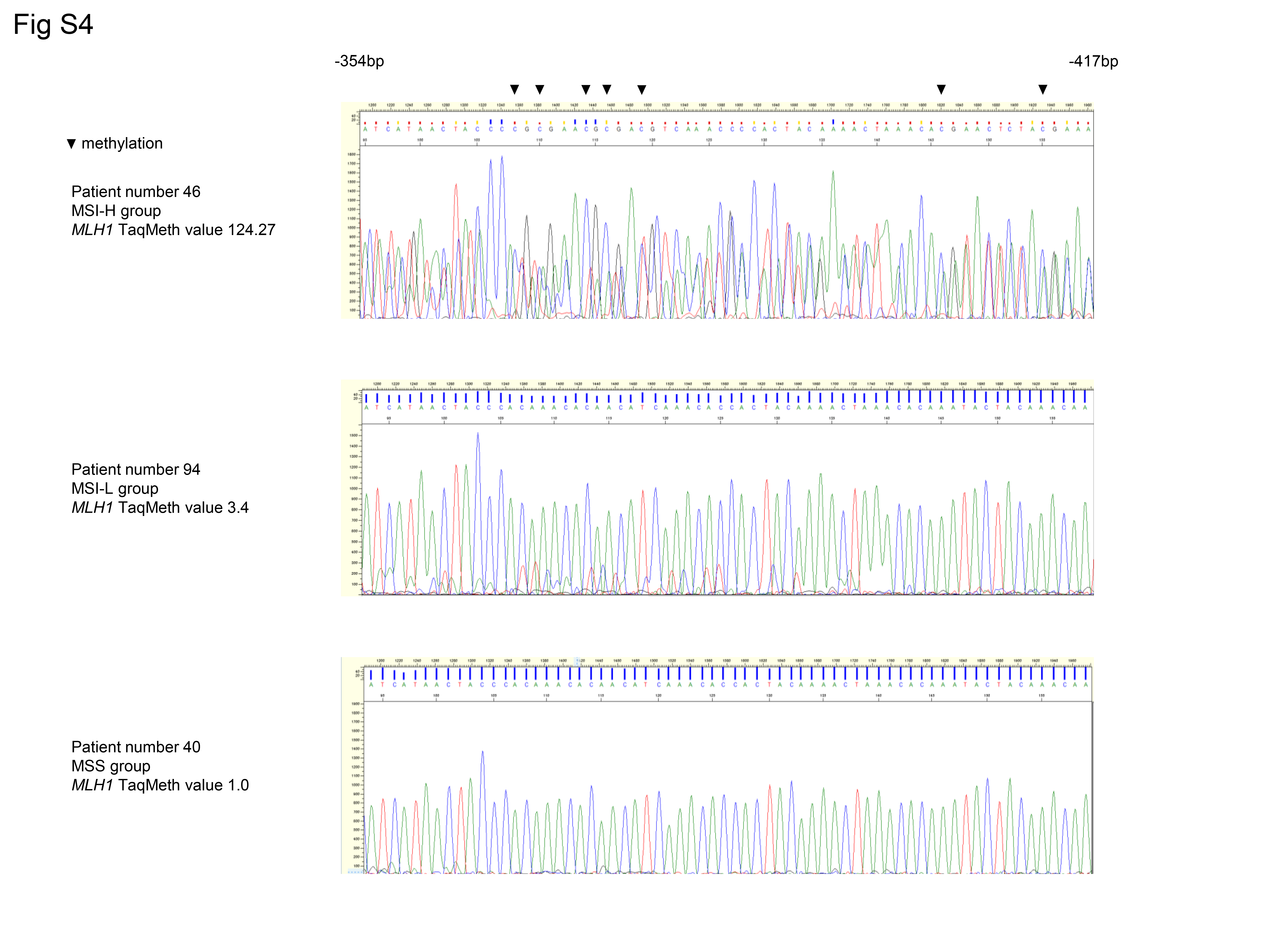

Supplement: S4 Fig — (TIF) [file pone.0260303.s004.tif]

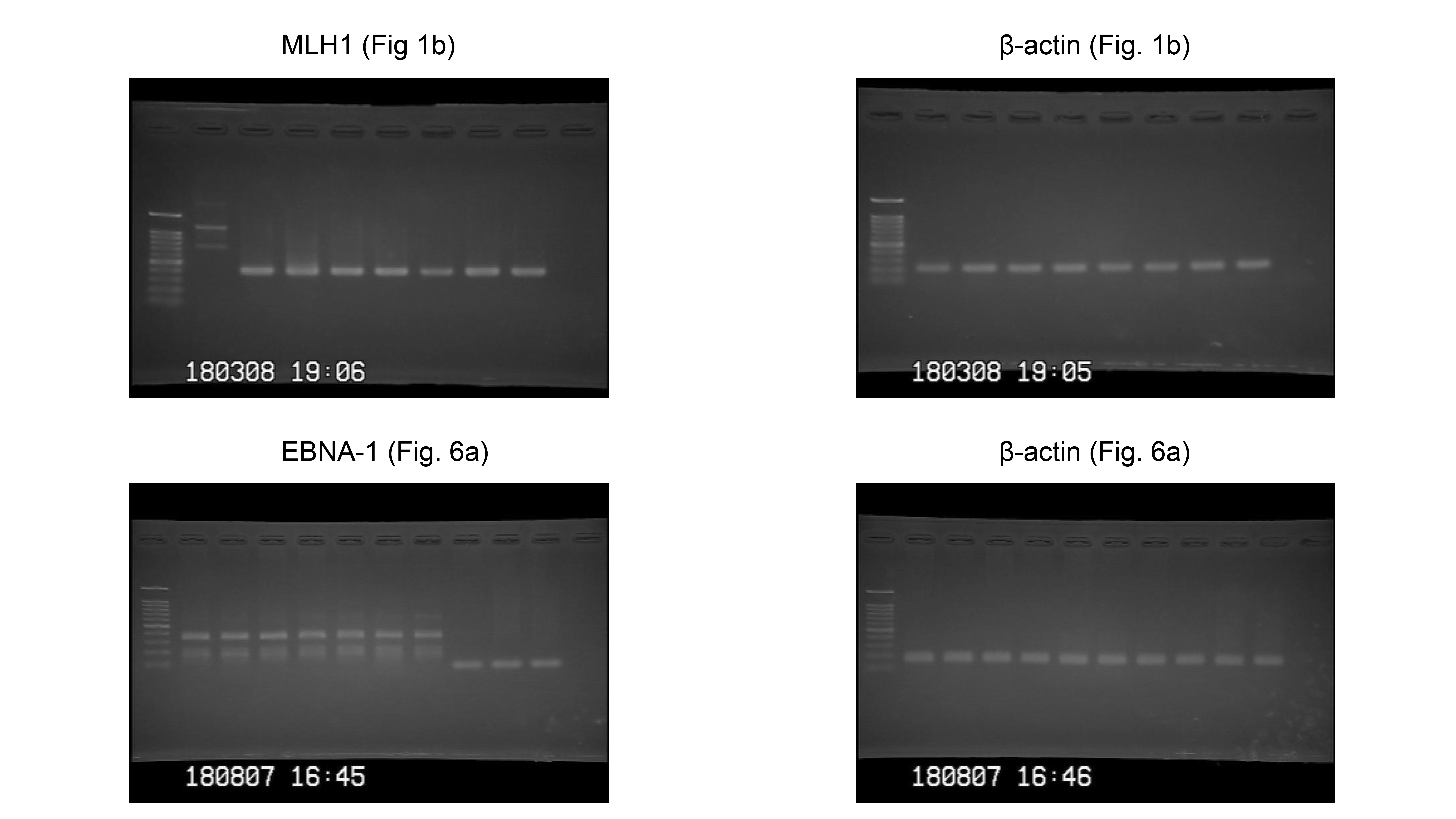

Supplement: S1 Raw images — (TIF) [file pone.0260303.s009.tif]
