# Supplementary material for: Haploinsufficiency by minute MutL homolog 1 promoter DNA methylation may represent unique phenotypes of microsatellite instability-gastric carcinogenesis
Source: PLoS One. 2021 Dec 22;16(12):e0260303. doi: 10.1371/journal.pone.0260303 (PMC8694418; doi:10.1371/journal.pone.0260303)
Supplement: Attachment — Submitted filename: Response to Reviewers.docx [file pone.0260303.s011.docx]

***PLoS One***

October 14, 2021

Dear Prof. Hiromu Suzuki, Editor

Thank you for inviting us to submit a revised draft of our manuscript entitled, “Haploinsufficiency by minute MutL homolog 1 promoter DNA methylation may represent unique phenotypes of microsatellite instability-gastric carcinogenesis” to PLoS One. We also appreciate the time and effort you and each of the reviewers have dedicated to providing insightful feedback on ways to strengthen our paper. Thus, it is with great pleasure that we resubmit our article for further consideration. We have incorporated changes that reflect the detailed suggestions you have graciously provided. We also hope that our edits and the responses we provide below satisfactorily address all the issues and concerns you and the reviewers have noted.

To facilitate your review of our revisions, the following is a point-by-point response to the questions and comments delivered in your letter.

Reviewer's Responses to Questions

Comments to the Author

1. If the authors have adequately addressed your comments raised in a previous round of review and you feel that this manuscript is now acceptable for publication, you may indicate that here to bypass the “Comments to the Author” section, enter your conflict-of-interest statement in the “Confidential to Editor” section, and submit your "Accept" recommendation.

Reviewer #1: All comments have been addressed

Reviewer #2: (No Response)

2. Is the manuscript technically sound, and do the data support the conclusions?

The manuscript must describe a technically sound piece of scientific research with data that supports the conclusions. Experiments must have been conducted rigorously, with appropriate controls, replication, and sample sizes. The conclusions must be drawn appropriately based on the data presented.

Reviewer #1: Yes

Reviewer #2: Yes

3. Has the statistical analysis been performed appropriately and rigorously?

Reviewer #1: Yes

Reviewer #2: Yes

4. Have the authors made all data underlying the findings in their manuscript fully available?

The PLOS Data policy requires authors to make all data underlying the findings described in their manuscript fully available without restriction, with rare exception (please refer to the Data Availability Statement in the manuscript PDF file). The data should be provided as part of the manuscript or its supporting information, or deposited to a public repository. For example, in addition to summary statistics, the data points behind means, medians and variance measures should be available. If there are restrictions on publicly sharing data—e.g. participant privacy or use of data from a third party—those must be specified.

Reviewer #1: Yes

Reviewer #2: Yes

5. Is the manuscript presented in an intelligible fashion and written in standard English?

PLOS ONE does not copyedit accepted manuscripts, so the language in submitted articles must be clear, correct, and unambiguous. Any typographical or grammatical errors should be corrected at revision, so please note any specific errors here.

Reviewer #1: Yes

Reviewer #2: Yes

6. Review Comments to the Author

Please use the space provided to explain your answers to the questions above. You may also include additional comments for the author, including concerns about dual publication, research ethics, or publication ethics. (Please upload your review as an attachment if it exceeds 20,000 characters)

Reviewer #1: (No Response)

Reviewer #2: The authors have addressed most of the comments. However, to avoid false positivity of qMSP, bisulphite sequencing of hMHL1 should be performed in selective samples in the haplo-MSI and low/high-MSI group

Thank you for your pertinent comments. Following reviewer's suggestion, we examined eight cases using direct sequencing. As a result, methylation could be detected in only 3 cases. The remaining 5 cases were MSI-L or MSS cases. Therefore, it was considered that the sensitivity to detect methylation of direct sequencing is limited to *MLH1* hypermethylation cases, and MSI-haplo can only be detected by real-time PCR.

The above contents have been added to Table and Discussion (P19, Line23-P20, Line 3).

7. PLOS authors have the option to publish the peer review history of their article (what does this mean?). If published, this will include your full peer review and any attached files.

If you choose “no”, your identity will remain anonymous but your review may still be made public.

Do you want your identity to be public for this peer review? For information about this choice, including consent withdrawal, please see our Privacy Policy.

Reviewer #1: No

Reviewer #2: No

In response to this revision, we asked the following doctor to give advice on the experiment, analysis and correct the rebuttal letter. Therefore, we would like to add the following one as co-author.

Yusuke Nie ^2^, Department of General-Pediatric-Hepatobiliary Pancreatic Surgery, Kitasato University School of Medicine, 1-15-1 Kitasato, Minami-ku, Sagamihara, Kanagawa 252-0375, Japan

Ippeita Araki ^1^, Department of Upper-gastrointestinal Surgery, Kitasato University School of Medicine, 1-15-1 Kitasato, Minami-ku, Sagamihara, Kanagawa 252-0375, Japan

We have found some typographical errors in the base sequence of Table S2. Therefore, we corrected it.

Again, thank you for giving us the opportunity to strengthen our manuscript with your valuable comments and queries. We have worked hard to incorporate your feedback and hope that these revisions persuade you to accept our submission.

Sincerely,

Hiroki Harada, MD, PhD

Keishi Yamashita, MD, PhD, FACS

Corresponding Author: Keishi Yamashita

Division of Advanced Surgical Oncology, Department of Research and Development Center for New Medical Frontiers, Kitasato University School of Medicine

1-15-1, Kitasato, Minami-ku, Sagamihara, KANAGAWA, 252-0374, JAPAN

E-mail: keishi23@med.kitasato-u.ac.jp

Tel: +81-42-778-8111 / Fax: +81-42-778-9556
